# Estradiol increases the sensitivity of ventral tegmental area dopamine neurons to dopamine and ethanol

**DOI:** 10.1371/journal.pone.0187698

**Published:** 2017-11-06

**Authors:** Bertha J. Vandegrift, Chang You, Rosalba Satta, Mark S. Brodie, Amy W. Lasek

**Affiliations:** 1 Department of Physiology and Biophysics, University of Illinois at Chicago, Chicago, Illinois, United States of America; 2 Department of Psychiatry, University of Illinois at Chicago, Chicago, Illinois, United States of America; Oregon Health and Science University, UNITED STATES

## Abstract

Gender differences in psychiatric disorders such as addiction may be modulated by the steroid hormone estrogen. For instance, 17β-estradiol (E2), the predominant form of circulating estrogen in pre-menopausal females, increases ethanol consumption, suggesting that E2 may affect the rewarding properties of ethanol and thus the development of alcohol use disorder in females. The ventral tegmental area (VTA) is critically involved in the rewarding and reinforcing effects of ethanol. In order to determine the role of E2 in VTA physiology, gonadally intact female mice were sacrificed during diestrus II (high E2) or estrus (low E2) for electrophysiology recordings. We measured the excitation by ethanol and inhibition by dopamine (DA) of VTA DA neurons and found that both excitation by ethanol and inhibition by dopamine were greater in diestrus II compared with estrus. Treatment of VTA slices from mice in diestrus II with an estrogen receptor antagonist (ICI 182,780) reduced ethanol-stimulated neuronal firing, but had no effect on ethanol-stimulated firing of neurons in slices from mice in estrus. Surprisingly, ICI 182,780 did not affect the inhibition by DA, indicating different mechanisms of action of estrogen receptors in altering ethanol and DA responses. We also examined the responses of VTA DA neurons to ethanol and DA in ovariectomized mice treated with E2 and found that E2 treatment enhanced the responses to ethanol and DA in a manner similar to what we observed in mice in diestrus II. Our data indicate that E2 modulates VTA neuron physiology, which may contribute to both the enhanced reinforcing and rewarding effects of alcohol and the development of other psychiatric disorders in females that involve alterations in DA neurotransmission.

## Introduction

Dysfunctions in dopaminergic (DA) neurotransmission contribute to many psychiatric disorders including mood disorders, schizophrenia, Parkinson’s disease, Tourette’s syndrome, and addiction [1–4]. The role of the DA system in psychiatric disorders has been established in animal models of these conditions and using human brain imaging and post-mortem tissue analysis [1, 4]. Interestingly, several psychiatric disorders also exhibit a gender bias in prevalence and severity [5]. Gender differences in the age of onset, prevalence, progression, and severity of symptoms associated with psychiatric disorders may be due to inherent sex differences in the organization of the DA system and/or modulated by sex hormones [6–8]. It is therefore important to understand the biological factors that underlie sex differences in DA neurotransmission to help guide the development of future treatments for psychiatric disorders that will be effective in both sexes.

The mesocorticolimbic DA system is critically involved in the development of addiction. This system encompasses the ventral tegmental area (VTA) DA neurons and their projections to the nucleus accumbens, amygdala, and prefrontal cortex [9–11]. VTA neurons are activated and release DA in their target areas in response to stimuli that are rewarding or novel [12], and release of dopamine by DA VTA neurons underlies the rewarding and reinforcing effects of drugs of abuse [13, 14]. The VTA is important for promoting alcohol consumption and reward [15, 16]. Rodents will self-administer ethanol directly into the VTA, suggesting that at least some of the reinforcing effects of ethanol are mediated through the VTA [16–18]. Acute administration of ethanol dose-dependently enhances the basal firing rate of rodent VTA DA neurons both *in vitro* [19, 20] and *in vivo* [21].

There are considerable gender differences in addiction, including differences in the amount of use, age of initiation, reasons for abuse, development of comorbidities, and severity of health problems [22]. Although more men abuse alcohol than women, the difference in the incidence of alcohol abuse is narrowing between genders [23, 24]. Women are more susceptible to the devastating health effects associated with alcohol abuse such as liver disease and neurotoxicity [25, 26]. Similar to other drugs of abuse such as cocaine, women also exhibit a greater escalation of alcohol use and are more likely to relapse in response to stress and anxiety [27]. Studies investigating differences in alcohol consumption during the normal human menstrual cycle are conflicting and indeterminate; thus, the precise role that the hormonal cycle has on alcohol intake in women is unknown [27]. However, steroid hormones, including estrogen, may play a role in sex differences in addiction and alcoholism [22, 28–30].

Estrogen, specifically 17β-Estradiol (E2), may affect the normal physiology of the VTA and the pathophysiology of disease states such as addiction. E2 modulates DA responses to psychostimulants [31–34] and increases the rewarding and reinforcing properties of cocaine and amphetamine [31, 35–41]. E2 alters mesolimbic DA transmission pre- and postsynaptically by increasing DA synthesis, reuptake, and release and changing receptor expression and activity [42–46]. Although much is known regarding the effects of E2 on behavioral and neurochemical responses to psychostimulants, the potential effects of E2 on behaviors related to alcohol abuse and the physiological responses of VTA DA neurons to ethanol are less well studied. In premenopausal women, alcohol consumption is positively correlated with serum E2 levels [47] and E2 supplementation in ovariectomized (OVX) mice increases ethanol-stimulated DA release in the prefrontal cortex [48]. Estrogen receptors are expressed in VTA neurons [49, 50]. However, the effects of E2 specifically on the dopamine- and ethanol-regulated responses of VTA neurons have not been examined. Here, we used gonadally intact female mice at different stages of the estrous cycle characterized by different circulating hormone levels and OVX mice supplemented with E2. We tested the firing of DA neurons in response to DA and ethanol. We demonstrate that E2 increases the sensitivity of VTA DA neurons to both ethanol and DA, indicating that sex differences in the DA system may be, in part, regulated hormonally. These results have implications for alcohol use disorder and other psychiatric diseases in women.

## Materials and methods

### Animals

Female C57BL/6J mice were used for all experiments. Mice were 8 weeks old when purchased from The Jackson Laboratories (Bar Harbor, ME, USA) and were tested between 10–14 weeks of age. Mice were group-housed under a 12 hour light-dark cycle in a temperature-and-humidity-controlled facility. Mice were treated in accordance with the National Institutes of Health *Guide for the Care and Use of Laboratory Animals*. All experimental procedures were approved by the Institutional Animal Care and Use Committee of the University of Illinois at Chicago and efforts were taken to alleviate suffering.

### Vaginal cytology

**Fig 1:**
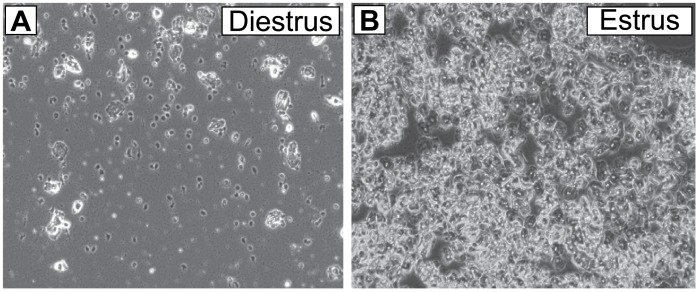
Representative images of vaginal smears from mice in estrus and diestrus. (A) Image showing a predominance of leukocytes in diestrus. (B) Image showing a predominance of anucleated epithelial cells in estrus.

Vaginal cytology was performed for at least two weeks in freely cycling females prior to performing experiments. A cotton swab was moistened with sterile water and gently inserted less than 1 mm into the vaginal opening and rotated. Immediately after removal from the vaginal opening, the swab was wiped on a microscope slide and the smear analyzed by bright field microscopy using an EVOS^®^ FL inverted microscope (Thermo Fisher Scientific). Estrous phase was determined based on vaginal cellular composition. For freely cycling mice, estrus vaginal composition was identified by an abundance of cornified epithelial cells, while diestrus II (herein referred to diestrus) was identified by the predominance of leukocytes in the smears [51](Fig 1). These two phases show large differences in circulating estrogen levels in mice, and are of substantially longer duration than either proestrus or metestrus/diestrus I [52]. For electrophysiology, the final smears were obtained 1–2 hours prior to euthanasia.

### OVX and E2 treatments

Mice were anesthetized with an intraperitoneal injection of ketamine (100 mg/kg) and xylazine (8 mg/kg). After shaving the hair on the back of the mouse, a small incision was made on the dorsal side, the uterine horn was pulled out of the abdominal cavity, and the ovary and associated fat pad were dissected away from the uterine horn with a cauterizer. The uterine horn was pushed back into the abdominal cavity. The incisions were closed with sterile sutures and wound clips. This procedure was performed bilaterally. Mice received an injection of meloxicam (2 mg/kg, subcutaneous) for analgesia immediately after surgery and 24 hours later. To confirm ovary removal, vaginal smears were taken daily from mice for 4–5 days and analyzed using bright field microscopy as described above. All OVX mice used in these experiments were no longer cycling through estrous, demonstrating successful ovary removal. After 10 days of recovery from surgery, the mice were treated for three days with 0.05 mL of sesame oil plus 10% ethanol vehicle (VEH) or E2 benzoate (EB) in VEH subcutaneously. For the first two days of treatment, mice were injected once daily with 0.2 μg EB or VEH, which resulted in serum E2 levels 4 hours after injection that were comparable to E2 levels in proestrus (S1 Fig). On the final day of treatment, the mice were injected with 1 μg EB or VEH one hour before sacrifice, which resulted in serum E2 levels 30 min after injection that were comparable to E2 levels in proestrus (S1 Fig).

### Preparation of brain slices

Brain slices containing the VTA were prepared for electrophysiology as previously described [53, 54]. Mice were briefly anesthetized with isoflurane prior to rapid removal of the brain. Using a vibratome and cold cutting solution, a tissue block containing the VTA and substantia nigra was cut into coronal sections of 400-μm thickness. Slices containing the VTA were placed in a chamber containing artificial cerebrospinal fluid (aCSF) flowing at a rate of 2 ml/min at 35°C. A small chamber (~0.5 ml) was used in these studies to allow the rapid application and washout of drug solutions in the bath. The slices were incubated for one hour in the aCSF before experiments were conducted. The composition of the aCSF was (in mM): NaCl 126, KCl 2.5, NaH2PO4 1.24, CaCl2 2.4, MgSO4 1.3, NaHCO3 26, glucose 11. The cutting solution composition was (in mM): KCl 2.5, CaCl2 2.4, MgSO4 1.3, NaHCO3 26, glucose 11, and sucrose 220. Both solutions were saturated with 95% O_2_/ 5% CO_2_ (pH = 7.4).

### Cell identification for electrophysiology

Putative DA cells (herein referred to as VTA DA neurons) were identified by anatomical location and electrophysiological characteristics. The VTA was visible in the slices as a grey area medial to the substantia nigra. White matter separated the VTA from the substanta nigra. DA neurons have been shown to have distinctive electrophysiological characteristics including broad action potentials (at least 2.5 msec), slow spontaneous firing rate (0.5–5 Hz), and a regular inter-spike interval. Only neurons located within the lateral VTA that conformed to previously established criteria for DA neurons were included [55]. The criteria used for the recorded VTA neurons are associated with DA-containing neurons projecting to the nucleus accumbens [56], although it should be noted that there is some controversy regarding the neurotransmitter identity of neurons with this electrophysiological profile [56–59]. We also tested baclofen on each of the neurons for the quinpirole studies, and 100% of them were inhibited by 1 μM baclofen. Baclofen has been shown to inhibit VTA DA neurons and not GABA neurons in the VTA [60].

### Drug administration

With the exception of the EB and VEH treatment of OVX mice, all drugs were administered during electrophysiological experiments. When ethanol and DA were added to the aCSF, a calibrated infusion pump from stock solutions was used. The final concentrations were calculated from the flow rate and stock solution concentrations (100 to 1000 times the desired final concentrations). The addition of the concentrated drugs to the flowing aCSF using the pumps was performed in such a way as to allow the drug solution to mix completely and reach the final desired concentration in the aCSF before reaching the slice. In some experiments, 10 μM ICI 182,780 was added to the microelectrode filling solution for pipette delivery, a technique that has been used in the past by our lab and others [61, 62].

### Extracellular recording

Spontaneous spike frequency of the putative DA neurons was measured using extracellular recordings; spike frequency (firing rate) was recorded and averaged over one-minute intervals. Recordings were made using micropipettes filled with 0.9% NaCl with tip resistances of 2 to 4 MΩ. Changes in firing rate were determined as peak percentage change relative to the baseline prior to drug administration.

### Statistical analysis

Averaged numerical values are presented as the mean ± SEM. Statistical comparisons were made using two-way ANOVA or two-way repeated measures ANOVA followed by Sidak’s multiple comparisons tests as appropriate (Origin, Originlab, Northampton, MA or Prism, Graphpad Software, Inc., La Jolla, CA). A *P* value of < 0.05 was considered significant.

## Results

### VTA neuron characteristics

A total of 121 VTA neurons were recorded in this study. Their initial basal firing rates ranged from 0.55 to 3.921 Hz. The mean firing rate was 1.996 ± 0.80 Hz. There were no significant differences in basal firing rate between estrus and diestrus of freely cycling mice (Estrus: 2.045 ± 0.15 Hz, Diestrus: 1.887 ± 0.16 Hz, t_53_ = 0.72, *P* = 0.48) or between EB- and vehicle-treated OVX mice (EB-treated: 1.953 ± 0.19 Hz, VEH-treated: 2.182 ± 0.13 Hz, t_50_ = 1.00, *P* = 0.09). All neurons conformed to the rate and patterns of DA VTA neurons as established in the literature and in this laboratory.

### Increased sensitivity of VTA DA neurons to inhibition by DA in diestrus

**Fig 2:**
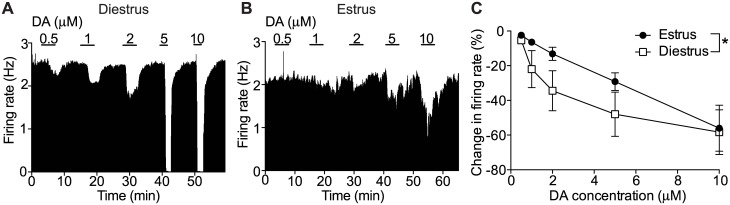
Increased sensitivity of VTA DA neurons to inhibition by DA during diestrus. Extracellular recordings of VTA DA neurons were obtained from mice in diestrus and estrus. The DA response was measured in putative VTA DA neurons with 0.5, 1, 2, 5, and 10 μM DA. (A-B) Representative rate meter graphs show the effects of DA during (A) diestrus and (B) estrus. (C) Concentration-response graph showing enhanced inhibition to DA in mice during diestrus (n = 9) compared with estrus (n = 9). *P < 0.05 by two-way ANOVA.

In addition to releasing DA onto their projection sites, DA neurons are also locally regulated by DA [63]. This DA-mediated autoregulation inhibits the firing of DA VTA neurons through actions at the dopamine D_2_ receptor (D2R) [64, 65]. In order to determine whether natural fluctuations of hormones affect responses to DA, we tested DA inhibition of VTA DA neurons from mice in diestrus and estrus. Representative vaginal smears of the estrus and diestrus phases of the cycle are shown in Fig 1. We found that VTA DA neurons from mice in diestrus exhibited a significantly enhanced inhibition by DA (0.5–10 μM) compared with VTA DA neurons from mice in estrus (Fig 2, n = 9 per group, two-way ANOVA, concentration: F_4,79_ = 11.05, *P* < 0.0001; phase: F_1,79_ = 4.75, *P* = 0.032; concentration x phase interaction: F_4, 79_ = 0.51, *P* = 0.73). For example, 5 μM DA decreased the firing rate by 48.0 ± 12.7% in neurons from mice in diestrus, while that same concentration of DA decreased the firing rate of neurons from mice in estrus by 29.6 ± 5.7%. To confirm that the enhanced inhibition observed during diestrus is mediated by D2/D3R stimulation, we tested the effect of quinpirole, a selective D2/D3R agonist. Quinpirole more potently inhibited DA neurons from mice in diestrus compared with estrus, similar to what we observed with DA (S2 Fig, n = 7 per group, two-way RM ANOVA, concentration: F_3, 36_ = 112.2, *P* < 0.0001; phase: F_1, 12_ = 6.39, *P* = 0.027; interaction: F _3, 36_ = 3.94, *P* = 0.016). Post-hoc multiple comparisons tests indicated a significant effect of 50 nM quinpirole (*P* = 0.001). In estrus, 50 nM quinpirole inhibited DA neurons by 36.6 ± 4.2%, while in diestrus, 50 nM quinpirole inhibited DA neurons by 72.9 ± 13.6%. These results indicate that DA autoinhibition of VTA DA neurons fluctuates during the estrous cycle and may be modulated by ovarian hormones.

### Increased sensitivity of VTA DA neurons to ethanol excitation in diestrus

**Fig 3:**
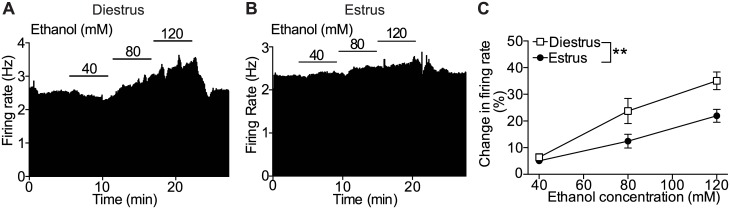
Increased sensitivity of VTA DA neurons to ethanol excitation during diestrus. Extracellular recordings of VTA DA neurons were obtained from mice in diestrus and estrus. The ethanol response was measured in VTA DA neurons with 40, 80, and 120 mM ethanol. Representative rate meter graphs show the effects of ethanol during (A) diestrus and (B) estrus. (C) Pooled concentration-response graph showing excitation by ethanol in mice during diestrus (n = 9) compared with estrus (n = 7). **P < 0.01 by two-way ANOVA.

We have established that ethanol directly increases the firing rate of VTA neurons of male rodents [53]. To determine if natural hormonal fluctuations in female mice affect the response of VTA DA neurons to ethanol, we tested excitation of VTA DA neurons from mice in diestrus and estrus by 40, 80, and 120 mM ethanol. VTA DA neurons from mice in diestrus showed significantly greater excitation by ethanol compared with neurons from mice in estrus (Fig 3, n = 7–9 per group, two-way ANOVA, concentration: F_2, 42_ = 27.74, *P* < 0.0001; phase: F_1, 42_ = 11.76, *P* = 0.0014; concentration x phase interaction: F_2, 42_ = 2.15, *P* = 0.13). For example, neurons from mice in estrus responded to 80 mM ethanol with a 12.4 ± 2.6% increase in firing rate, whereas in neurons from mice in diestrus, the same concentration of ethanol produced a 23.7 ± 4.7% increase in firing rate. These results indicate that ethanol more potently excites VTA DA neurons from female mice during diestrus compared with estrus, demonstrating that different hormonal states affect the response to ethanol.

### E2 enhances responses of VTA DA neurons to DA and ethanol

**Fig 4:**
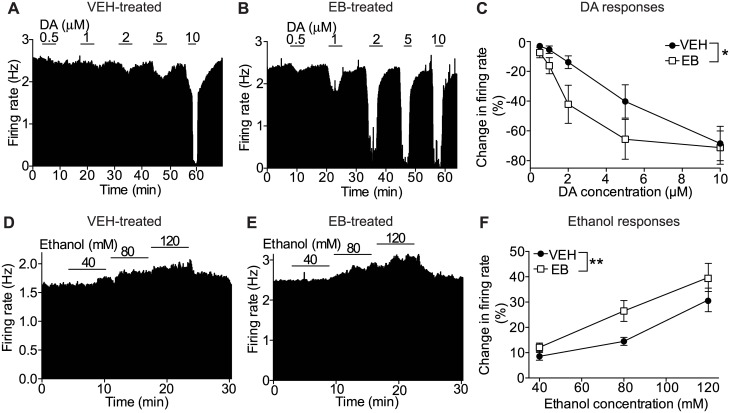
E2 enhances DA inhibition and ethanol excitation of VTA DA neurons. Extracellular recordings were obtained from VTA DA neurons in ovariectomized (OVX) mice previously treated with 17β-estradiol-3-benzoate (EB) or vehicle (VEH) (A-B) Representative rate meter graphs showing response to 0.5–10 μM DA of a VTA DA neuron from a mouse treated with VEH (A) or EB (B). (C) DA responses in VTA DA neurons treated with 0.5–10 μM DA. Concentration-response graph shows enhanced inhibition by DA in OVX mice treated with EB compared with VEH (n = 9 per group). (D-E) Representative rate meter graphs showing response to 40–120 mM ethanol of a VTA DA neuron from a mouse treated with VEH (D) or EB (E). (F) Ethanol responses in VTA DA neurons treated with 40, 80, and 120 mM ethanol. Pooled concentration-response graph shows enhanced excitation to ethanol in OVX mice treated with EB (n = 13) compared with VEH (n = 12). *P < 0.05, **P < 0.01 by two-way ANOVA.

Since E2 levels are higher in diestrus compared with estrus [52], we hypothesized that the increased responses of VTA neurons to DA and ethanol during diestrus may be due to an effect of elevated E2. To more directly test this hypothesis, mice were OVX to remove circulating hormones produced by the ovaries and treated systemically with daily EB or VEH injections for three days prior to sacrifice. Brain slices were prepared one hour after the last EB or VEH injection and VTA neurons tested for electrophysiological responses to DA and ethanol. VTA DA neurons in OVX mice treated with EB demonstrated a significantly increased sensitivity to DA inhibition (0.5–10 μM) compared with neurons from OVX VEH-treated mice (Fig 4, n = 9 per group, concentration: F_4, 80_ = 19.05, *P* < 0.0001; treatment: F_1, 80_ = 6.41, *P* = 0.013; concentration x treatment interaction: F_4, 80_ = 0.88, *P* = 0.48). For example, 5 μM DA decreased the firing rate by 65.6 ± 13.4% in neurons from EB-treated mice, while the decrease in firing rate in neurons from VEH-treated mice was 40.2 ± 11.2%. This result indicates that E2 pretreatment increases the sensitivity of VTA DA neurons to inhibition by DA.

We next tested for the effect of EB treatment on the excitation of VTA DA neurons by ethanol. Neurons from OVX mice treated with EB showed a significantly enhanced ethanol-induced (40–120 mM) excitation compared with neurons from OVX VEH-treated mice (Fig 4, n = 12–13, two-way ANOVA, concentration: F_2, 69_ = 24.83, *P* < 0.0001; treatment: F _1, 69_ = 7.81, *P* = 0.0067; concentration x treatment interaction: F_2, 69_ = 0.71, *P* = 0.49). As an example, neurons from EB-treated mice responded to 80 mM ethanol with an increase in firing rate of 26.5 ± 4.2%, whereas neurons from VEH-treated mice responded to ethanol with an increase in firing rate of 14.5 ± 1.6%. Because GABAergic neurotransmission in the VTA may regulate the firing of VTA DA neurons [66], we also tested whether the enhanced ethanol excitation in neurons from EB-treated OVX mice was affected by GABA antagonists (concurrent administration of bicuculline and CGP35348, 10 μM each). In the presence of GABA antagonists, the response of VTA DA neurons to ethanol in OVX VEH- or EB-treated mice was similar to what we observed in the absence of these antagonists, suggesting that the increase in ethanol excitation elicited by EB is independent of GABA neurotransmission in the VTA (S3 Fig).

### Estrogen receptors in the VTA acutely regulate the sensitivity of DA neurons to ethanol but not DA

**Fig 5:**
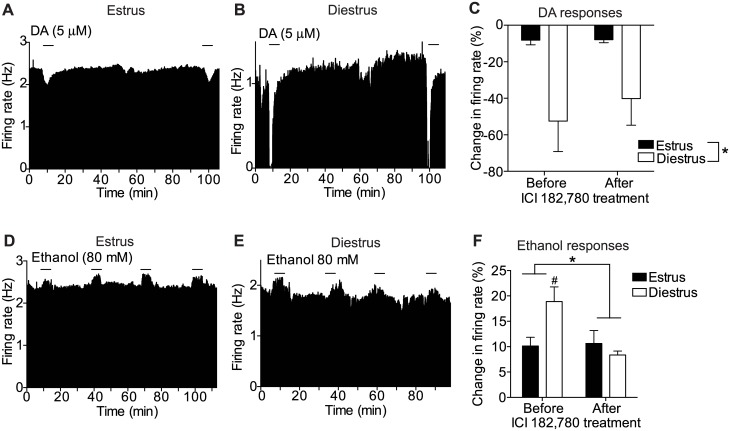
Estrogen receptors in the VTA acutely regulate the sensitivity of DA neurons to ethanol but not DA. (A-B) Representative rate meter graphs showing response to 5 μM DA of a VTA DA neuron from a mouse in estrus (A) or diestrus (B) recorded before and during treatment with ICI 182,780. (C) Response to 5 μM DA of VTA DA neurons from mice in estrus (n = 6) or diestrus (n = 6) before and after treatment of slices containing the VTA with ICI 182,780. There was no change in DA inhibition with ICI 182,780 treatment, but there was a significant effect of cycle phase (*P < 0.05). (D-E) Representative rate meter graphs showing response to 80 mM ethanol of a VTA DA neuron from a mouse in estrus (D) or diestrus (E) recorded before and during treatment with ICI 182,780. (F) Response to 80 mM ethanol of VTA DA neurons from mice in estrus (n = 6) or diestrus (n = 6) before and after treatment of slices containing the VTA with ICI 182,780. The enhanced VTA DA neuron excitation by ethanol that was observed in mice in diestrus was decreased after treatment with ICI 182,780, whereas the ethanol response of VTA DA neurons from mice in estrus was not altered by ICI 182,780. *P < 0.05 by two-way RM ANOVA. ^#^*P* < 0.05 between mice in estrus and diestrus before ICI 182,780 treatment by post-hoc Sidak’s multiple comparisons test.

Estrogen receptors are present in the VTA and are expressed in DA neurons [49, 50, 67, 68]. To determine if estrogen receptors in the VTA regulate the increased sensitivity of VTA DA neurons to ethanol and DA, we treated brain slices containing the VTA with the estrogen receptor (ERα and ERβ) antagonist ICI 182,780 and measured the electrophysiological responses to DA and ethanol in neurons from mice in estrus and diestrus. ICI 182,780 was delivered via the recording pipette, a method we have used successfully in the past [61]. ICI 182,780 did not affect inhibition by DA in either estrus or diestrus. However, similar to the effect shown above in Fig 2, there was a significant main effect of estrous cycle phase, indicating enhanced DA inhibition in neurons from mice in diestrus (Fig 5, n = 6 per group, two-way RM ANOVA, treatment: F_1,10_ = 1.75, *P* = 0.22; phase: F_1,10_ = 6.45, *P* = 0.029; treatment x phase interaction: F_1,10_ = 1.62, *P* = 0.23). The lack of effect of ICI 182,780 on inhibition of VTA DA neurons by DA suggests that estrogen receptors do not acutely affect DA inhibition, or function in a different brain region to modulate this effect.

In contrast, ethanol excitation of VTA DA neurons from mice in diestrus was decreased after treatment with ICI 182,780 (Fig 5, n = 6 per group, two-way RM ANOVA, treatment: F_1, 10_ = 7.29, *P* = 0.022; phase: F_1, 10_ = 1.83, *P* = 0.21; treatment x phase interaction: F_1, 10_ = 8.79, *P* = 0.014). Post-hoc multiple comparisons testing demonstrated a significant difference in ethanol-stimulated firing in VTA DA neurons from mice in diestrus compared with estrus before treatment with ICI 182,780 (*P* = 0.018), consistent with results shown in Fig 3. This difference in ethanol-stimulated firing between these two phases of the estrous cycle was abolished after allowing 90 minutes for ICI 182,780 to diffuse from the recording pipette onto the slice. Neurons from mice in the diestrus phase responded to 80 mM ethanol with an 18.8 ± 7.1% increase in firing rate at the beginning of the experiment, but after the 90 minute of pipette delivery of ICI 182,780, the response to 80 mM ethanol was increased by only 8.4 ± 1.9%. As a control, we also tested if inclusion of DMSO vehicle in the recording pipette would change the response to ethanol of VTA DA neurons from diestrus mice over the same 90-minute period. There was no change in ethanol-stimulated firing over time with DMSO vehicle in the pipette (t_4_ = 0.94, *P* = 0.4, data not shown), indicating that the decrease in ethanol response from mice in diestrus after incubation with ICI 182,780 is not consequence of time, DMSO, or repetition of ethanol administration. In addition, the response to ethanol of VTA DA neurons from mice in estrus was not altered by ICI 182,780. At the beginning of the experiment, neurons from mice in the estrus phase responded to 80 mM ethanol with an increase of firing rate of 10.1 ± 4.2%; after 90 minutes of the pipette delivery of ICI 182,780, neurons from the estrus phase responded with an 11.5 ± 5.6% increase in firing rate. These results demonstrate that estrogen receptors in the VTA acutely regulate the enhancement of ethanol stimulation in the diestrus phase, when E2 levels are higher than in estrus.

## Discussion

Our results show that E2 modulates the response of VTA DA neurons to ethanol and DA. Here we tested the ethanol and DA responses of VTA neurons in both OVX and gonadally intact female mice. We found that elevated E2 corresponded with enhanced responses in VTA neurons. This was observed in neurons in OVX mice treated with E2 and in mice in diestrus, where serum E2 levels have been reported to reach ~6 pg/ml [52] compared with neurons from OVX mice and mice in estrus, where serum E2 levels have been estimated to be less than 0.3 pg/ml [52]. The responses to ethanol and DA of VTA DA neurons in mice in estrus (when circulating E2 levels are low) are comparable to responses that we have observed previously in male C57BL6/J mice, providing additional evidence that E2 increases the sensitivity of VTA DA neurons to ethanol and DA [69, 70]. Although we did not measure responses of VTA DA neurons during proestrus, when serum E2 levels in mice are slightly higher (~8 pg/mL) than in diestrus, we would expect that VTA DA neurons from mice in proestrus would behave in a similar manner to neurons from mice in diestrus in response to DA and ethanol. We also demonstrated that the enhanced ethanol excitation, but not the enhanced DA inhibition, observed in VTA neurons from mice in diestrus is reversed after acute treatment with ICI 182,780, an antagonist of the estrogen receptors ERα and ERβ.

Interestingly, we did not observe differences in basal firing rates of VTA neurons between estrus and diestrus from freely cycling or between EB- and VEH-treated OVX mice. Other studies have reported differences in basal firing activity according to the estrus phase of intact rodents with single-unit extracellular *in vivo* measurements. Basal firing rate and bursting were found to be greater in estrus compared with diestrus [71, 72]. One potential explanation for this discrepancy is that we recorded from *in vitro* brain slices and regulatory inputs that modulate these changes in basal firing properties may be masked or missing in our slice preparation. During slice preparation, the excitatory inputs responsible for burst firing in the VTA are disrupted [73].

DA inhibition was enhanced in VTA neurons from gonadally intact mice in diestrus and in OVX mice treated with EB. Inhibition of firing by D2R activation is mediated by potassium channels activated by the βγ subunit of G proteins [74]. The alterations of responses to DA by E2 that we observed might be due to a change in D2R expression, trafficking, G protein coupling, or other downstream signaling components. Future studies will address which of these possibilities contribute to the E2 enhancement of DA inhibition.

Acute in vitro treatment with the ER antagonist, ICI 182,780, did not alter the inhibitory DA response of VTA DA neurons from mice in diestrus or estrus. While it is possible that the increased DA inhibition observed in VTA neurons from mice in diestrus is not due to increased E2 levels, this seems unlikely. We found that treatment of OVX mice with E2 resulted in increased inhibition of VTA DA neurons by DA when compared with VEH-treated mice, similar to differences in DA responses of DA VTA neurons from mice in diestrus (higher E2 levels) compared with estrus (lower E2 levels); these observations support the argument that E2 is responsible for increasing the sensitivity of VTA DA neurons to inhibition by DA in intact females. It seems more likely that the alteration of D2 sensitivity is caused by E2-induced changes in DA responsiveness in the VTA elicited prior to performing the electrophysiology. For instance, acute ICI 182,780 treatment would not be expected to reverse the increased DA sensitivity in high estrogen states if E2 acted prior to slice preparation to change the expression of genes that regulate D2R signaling or trafficking (i.e. the classical or genomic action of ERs). The timing of E2 injections in our experiments is sufficiently long to permit genomic changes to occur, since we pre-treated the mice for three days prior to performing the experiment. Presumably the rise in E2 levels in mice in diestrus before we prepared slices is also long enough to elicit genomic changes that could increase the response to DA. Alternatively, the effect of E2 in vivo might indirectly increase the response to DA by employing a polysynaptic mechanism, for example. Finally, it is possible that the lack of effect of acute ICI 182,780 on DA inhibition is merely the result of E2 acting on ERs outside of the VTA to change responses to DA in the VTA. Future experiments need to be performed to distinguish among these possibilities.

We found that ethanol excitation was enhanced in VTA neurons from gonadally intact mice in diestrus and OVX mice treated with EB. Increased ethanol-induced excitation of DA neurons during elevated E2 states would lead to increased DA release, and is predicted to enhance ethanol reward [75, 76]. Ethanol excites VTA neurons *in vivo* [21] and directly increases the spontaneous firing rate of dissociated VTA DA neurons [53]. Ethanol excitation is retained under conditions that block synaptic transmission in brain slices containing the VTA [19] and in dissociated VTA DA neurons [53].

The increased sensitivity to ethanol of VTA DA neurons in mice in diestrus and in OVX mice treated with EB suggests that behaviors related to the rewarding and reinforcing properties of ethanol will be enhanced when circulating E2 levels are high. Indeed, several studies have demonstrated that OVX mice and rats treated with E2 consume more ethanol compared with vehicle-treated OVX mice [77–83]. In addition, female rodents tend to consume more ethanol than male rodents [27, 84–86]. By re-examining our published studies of the ethanol responses of VTA DA neurons in male C57BL/6J mice [69, 70], we observed that the concentration-response to ethanol is similar between males and estrus females (when serum E2 concentrations are low). This might suggest that mice in diestrus or proestrus would consume more ethanol than male or estrus mice. However, several studies have found that ethanol consumption does not change throughout the estrous cycle in rats [85, 87, 88] and we have also not observed differences in ethanol consumption throughout the estrous cycle in freely cycling mice (R. Satta and A.W. Lasek, manuscript in revision). One reason for this may be that timing is critical when testing for behavioral differences. The estrous cycle in mice and rats is very short (~4 days) and within a specific cycle phase, hormone levels can change rapidly. Indeed, Roberts *et al* did find cycle phase differences in ethanol self-administration in rats when their cycles were synchronized using an agonist to the gonadotropin-releasing hormone receptor [88]. The sex difference in ethanol consumption observed in mice could be due to higher levels of E2 in females contributing to increased sensitivity of VTA DA neurons to excitation by ethanol and perhaps higher levels of drinking. This remains to be determined, but is an important area for future investigation.

The ethanol-induced excitation of VTA DA neurons was assessed using high concentrations (40–120 mM). Although the concentrations used here were relatively high, we and others have found that 4 hours of access to 20% ethanol in the “drinking in the dark” protocol routinely leads to blood ethanol concentrations of ~35–43 mM, with females at the high end of that range [54, 86] (R. Satta and A.W. Lasek, manuscript in revision). In addition, the *in vitro* brain slice preparation may eliminate innervation from other brain regions that may contribute to the VTA response to ethanol, such as the serotonergic innervation from the Raphe nuclei [89]. Without these synaptic inputs, the VTA DA sensitivity to ethanol may be suppressed.

We have demonstrated previously that ethanol-induced excitation of VTA DA neurons in our recordings is unaltered by GABAergic or glutamatergic antagonists [90]. In the present study, we also found that the enhancement of ethanol-stimulated firing of VTA DA neurons by E2 is not altered by GABA receptor antagonists. Although E2 has been shown to inhibit GABA release in the striatum and hippocampus and alter expression of GABA-A receptor subunits [91–93], these data suggest that the ability of E2 to increase ethanol-induced excitation of VTA DA neurons is likely not due to changes in GABA neurotransmission elicited by E2.

In addition to finding enhanced ethanol-induced excitation of VTA DA neurons from mice in diestrus compared with estrus, we observed that treatment of slices acutely with ICI 182,780 reduced ethanol excitation during diestrus, but not estrus. This suggests that ongoing signaling by membrane-bound ERs in the VTA is responsible for the enhanced excitation by ethanol, rather than the effects of ERs on transcription. These observations from mice in diestrus are consistent with the finding that treatment of mice with E2 increases excitation of VTA DA neurons by ethanol. ICI 182,780 is considered to be a ‘‘pure” ERα/β antagonist [94], but was recently found to act as an agonist at the G protein coupled ER, GPER1 [95]. We believe that the ability of ICI 182,780 to block ethanol-induced excitation of VTA DA neurons during diestrus is likely due to its inhibition of ERα and/or ERβ because activation of GPER1 by ICI 182,780 would have an estrogenic action rather than reversal of the estrogen effect on ethanol potency that we observed. The mechanism of action of ICI 182,780 on inhibiting ERα activity has been described on multiple levels including interfering with the formation of ERα dimers, inhibiting nuclear translocation, increasing protein degradation, and blocking rapid signaling by membrane-bound ERα [94, 96–98]. ICI 182,780 also blocks ERβ-mediated transcriptional activity [99] and inhibits E2-mediated changes in amphetamine-stimulated striatal DA release in female rats [100]. The acute reduction of ethanol excitation by ICI 182,780 observed in VTA neurons from mice in the diestrus phase suggests that the increased sensitivity to ethanol during diestrus may be due to active engagement of ERs by E2 in the slice preparation. The E2-mediated enhancement of ethanol-stimulated firing of VTA DA neurons might therefore be due to rapid signaling effects mediated by membrane-bound ERs in the VTA, rather than genomic or transcriptional actions of these receptors, although there is cross-talk between genomic and membrane E2 action observed in regions such as the hippocampus and hypothalamus that could be responsible for the enhancement of ethanol excitation of VTA DA neurons [101, 102]

We have not investigated which ER subtypes are involved in modulating DA and ethanol sensitivity of VTA DA neurons. We predict that E2 is acting through ERα or ERβ. ERβ expression has been investigated using a transgenic reporter mouse [49] and specific antibodies [103] and is expressed in VTA DA and non-DA neurons. We have also performed fluorescent immunostaining for ERα in mouse VTA and have found that ERα is expressed in both VTA DA and non-DA neurons (R. Satta and A.W. Lasek, unpublished results). It is possible that the ability of E2 to affect responses of VTA DA neurons to ethanol and DA are through actions on either ERα and/or ERβ. Overall the data presented here indicate that elevated estrogen states, in both gonadally intact and E2-supplemented OVX mice, enhance ethanol excitation and dopamine inhibition. Future studies will investigate which ERs play a role in these electrophysiological responses of the VTA and the molecular mechanisms for the enhanced ethanol excitation and dopamine inhibition.

## Supporting information

S1 Fig Serum E2 levels in OVX female mice treated with EB. Mice were treated with the indicated doses of EB (0.2 or 1 μg) and whole blood collected at 30 min or 4 hours after injection. Serum was analyzed for E2 levels using an enzyme-linked immunoassay kit from Cayman Chemical. For comparison, serum from mice in proestrus was also analyzed. Oil, n = 8; at 30 min: EB 0.2, n = 2; EB 1, n = 2; at 4 hours: EB 0.2, n = 5; EB 1, n = 6; proestrus, n = 3.
(TIFF)

S2 Fig Enhanced inhibition by quinpirole in VTA DA neurons in diestrus mice. Extracellular recordings were made from VTA slices from mice in estrus or diestrus. Shown are the concentration-response curves showing quinpirole inhibition of VTA DA neurons (n = 7 per group). There was a significant main effect of concentration (F_3, 36_ = 112.2, *P* < 0.0001), estrous cycle phase (F_1, 12_ = 6.39, *P = 0.027), and a significant interaction (F_3, 36_ = 3.94, *P* = 0.016) by two-way RM ANOVA. Post-hoc Sidak’s multiple comparisons tests indicated a significant difference between estrus and diestrus at 50 nM quinpirole, indicated by two asterisks (**P < 0.01).
(TIFF)

S3 Fig GABA antagonists do not alter the ethanol-stimulated firing of DA neurons in OVX mice treated with E2. Extracellular recordings were made from VTA slices from OVX mice treated with E2 or VEH. The GABA antagonists bicuculline and CGP35348 (10 μM each) were administered concurrently to the slices. Shown is the ethanol concentration-response graph in the presence or absence of the GABA antagonists (n = 4–5, two-way ANOVA, For vehicle treated: ethanol concentration: F_2, 20_ = 10.50, *P* < 0.0001; treatment: F _1,20_ = 0.02, *P* = 0.90; For E2 treated: ethanol concentration: F_2, 26_ = 16.05, *P* < 0.0001; treatment: F _1,26_ = 0.11, *P* = 0.75).
(TIFF)
