# Supplementary figures and images for: Estradiol increases the sensitivity of ventral tegmental area dopamine neurons to dopamine and ethanol
Source: PLoS One. 2017 Nov 6;12(11):e0187698. doi: 10.1371/journal.pone.0187698 (PMC5673180; doi:10.1371/journal.pone.0187698)

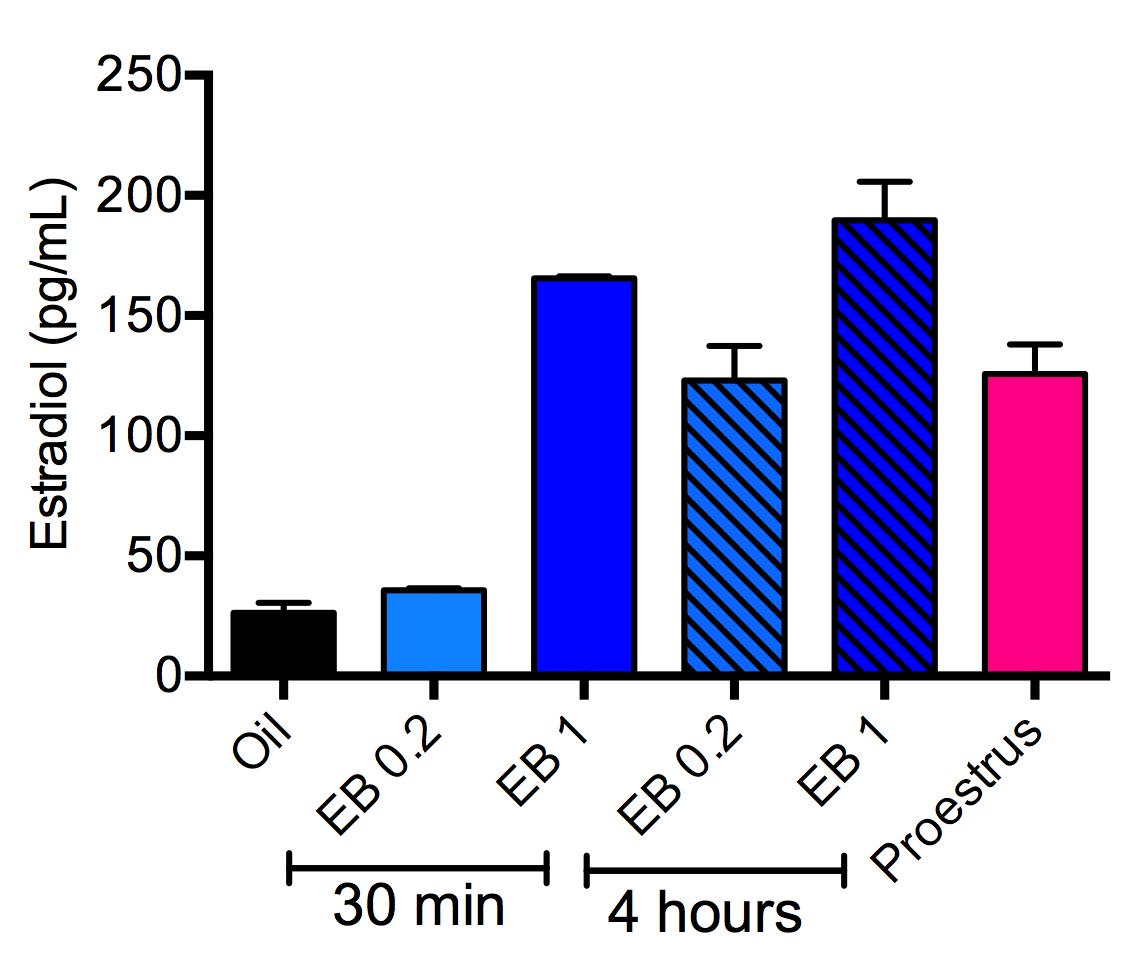

Supplement: S1 Fig — Mice were treated with the indicated doses of EB (0.2 or 1 μg) and whole blood collected at 30 min or 4 hours after injection. Serum was analyzed for E2 levels using an enzyme-linked immunoassay kit from Cayman Chemical. For comparison, serum from mice in proestrus was also analyzed. Oil, n = 8; at 30 min: EB 0.2, n = 2; EB 1, n = 2; at 4 hours: EB 0.2, n = 5; EB 1, n = 6; proestrus, n = 3. (TIFF) [file pone.0187698.s001.tiff]

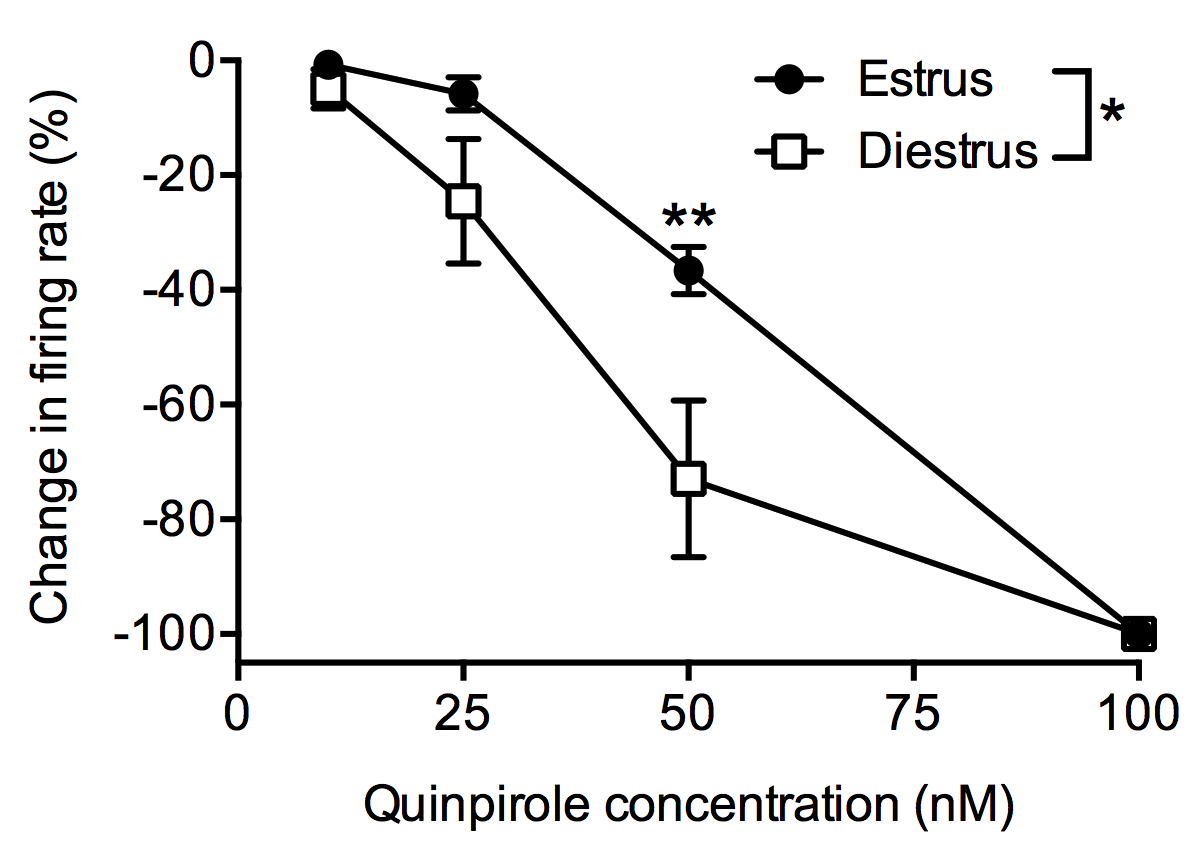

Supplement: S2 Fig — Extracellular recordings were made from VTA slices from mice in estrus or diestrus. Shown are the concentration-response curves showing quinpirole inhibition of VTA DA neurons (n = 7 per group). There was a significant main effect of concentration (F3, 36 = 112.2, P < 0.0001), estrous cycle phase (F1, 12 = 6.39, *P = 0.027), and a significant interaction (F3, 36 = 3.94, P = 0.016) by two-way RM ANOVA. Post-hoc Sidak’s multiple comparisons tests indicated a significant difference between estrus and diestrus at 50 nM quinpirole, indicated by two asterisks (**P < 0.01). (TIFF) [file pone.0187698.s002.tiff]

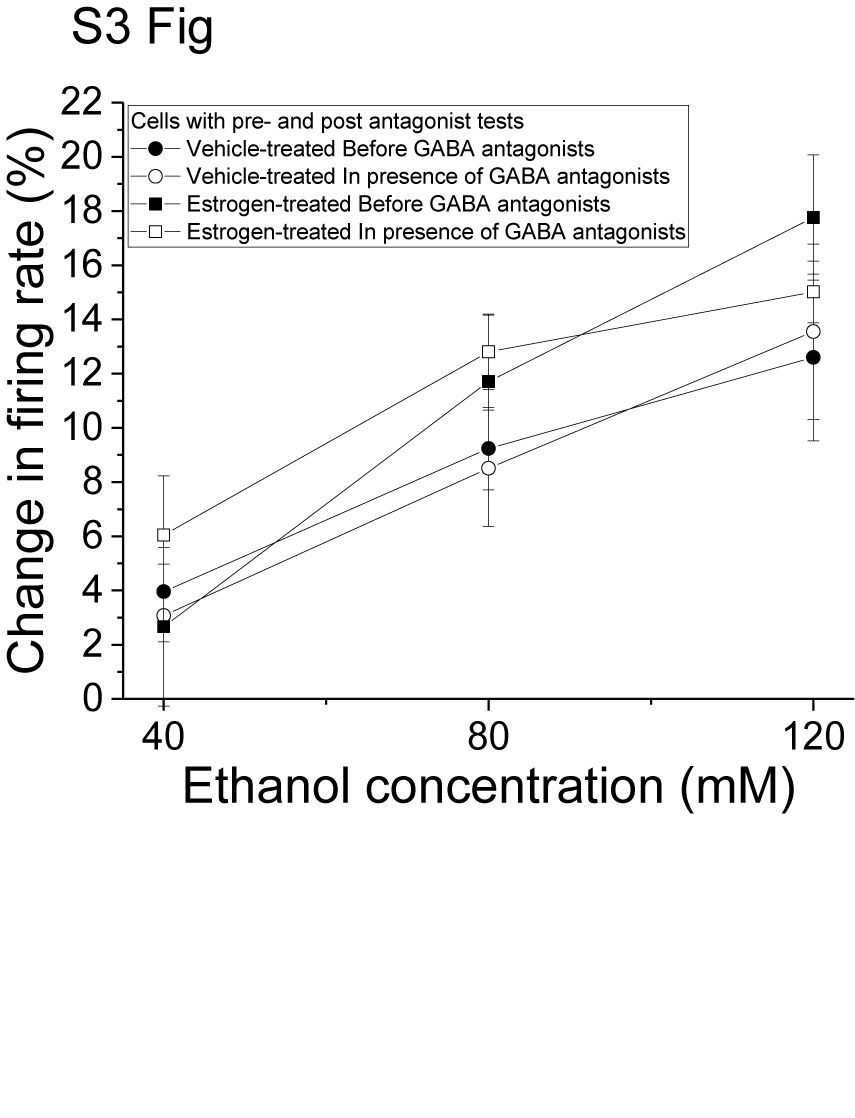

Supplement: S3 Fig — Extracellular recordings were made from VTA slices from OVX mice treated with E2 or VEH. The GABA antagonists bicuculline and CGP35348 (10 μM each) were administered concurrently to the slices. Shown is the ethanol concentration-response graph in the presence or absence of the GABA antagonists (n = 4–5, two-way ANOVA, For vehicle treated: ethanol concentration: F2, 20 = 10.50, P < 0.0001; treatment: F 1,20 = 0.02, P = 0.90; For E2 treated: ethanol concentration: F2, 26 = 16.05, P < 0.0001; treatment: F 1,26 = 0.11, P = 0.75). (TIFF) [file pone.0187698.s003.tiff]
